# Non-adherence to antihypertensive pharmacotherapy in Buea, Cameroon: a cross-sectional community-based study

**DOI:** 10.1186/s12872-018-0888-z

**Published:** 2018-07-24

**Authors:** Nkengla Menka Adidja, Valirie Ndip Agbor, Jeannine A. Aminde, Calypse A. Ngwasiri, Kathleen Blackett Ngu, Leopold Ndemnge Aminde

**Affiliations:** 1 Faculty of Health Sciences, University of Buea, Buea, Cameroon; 2 Djeleng Sub-divisional Hospital, Bafoussam, Cameroon; 3 Ibal Sub-divisional Hospital, Oku, Cameroon; 4 Faculty of Medicine & Biomedical Sciences, University of Yaoundé 1, Yaoundé, Cameroon; 5 Etoug-Ebe Baptist Hospital, Yaoundé, Cameroon; 6 Bamendjou District Hospital, Bamendjou, Cameroon; 7 Clinical Research Education, Networking & Consultancy (CRENC), Douala, Cameroon; 8 Faculty of Medicine, School of Public Health, The University of Queensland, Brisbane, Australia

**Keywords:** Hypertension, Medication adherence, Non-adherence, Morisky scale, Cameroon

## Abstract

**Background:**

Hypertension is a challenging public health problem with a huge burden in the developing countries. Non-adherence to antihypertensive treatment is a big obstacle in blood pressure (BP) control and favours disease progression to complications. Our objectives were to determine the rate of non-adherence to antihypertensive pharmacotherapy, investigate factors associated with non-adherence, and to assess the association between non-adherence and BP control in the Buea Health District (BHD), Cameroon.

**Methods:**

A community-based cross-sectional study using stratified cluster sampling was conducted in the BHD from November 2013 – March 2014. Eligible consenting adult participants had their BP measured and classified using the Joint National Committee VII criteria. The Morisky medication adherence scale was used to assess adherence to BP lowering medication. Multivariable logistic regression models were used to predict non-adherence.

**Results:**

One hundred and eighty-three participants were recruited with mean age of 55.9 years. Overall, 67.7% (95% CI: 59.8–73.6%) of participants were non-adherent to their medications. After adjusting for age, sex and other covariates, forgetfulness (aOR = 7.9, 95%CI: 3.0–20.8), multiple daily doses (aOR = 2.5, 95%CI: 1.2–5.6), financial constraints (aOR = 2.8, 95%CI: 1.1–6.9) and adverse drug effects (aOR = 7.6, 95%CI: 1.7–33.0) independently predicted non-adherence to anti-hypertensive medication. BP was controlled in only 21.3% of participants and was better in those who were adherent to medication (47.5% versus 8.2%, *p* <  0.01).

**Conclusion:**

At least two of every three hypertensive patients in the Buea Health District are non-adherent to treatment. Forgetfulness, multiple daily doses of medication, financial constraints and medication adverse effects are the major predictors of non-adherence in hypertensive patients. These factors should be targeted to improve adherence and BP control, which will contribute to stem hypertension-related morbidity and mortality.

## Background

Hypertension is an important public health challenge worldwide. In 2010, the global prevalence of hypertension was estimated at 1.39 billion representing 31.1% in the adult population [1]. This implies one in three adults suffer from hypertension. Low- and middle-income countries are more affected than high income countries [2]. Compared with Caucasians, Africans with hypertension have lower hypertension control rates and higher prevalence of hypertension-related complications [3]. The prevalence of hypertension in Africa was estimated at 30.8% in 2014 [4]. Studies done in specific populations in Cameroon have equally revealed prevalence rates as high as 37.8% [5] and 57.3% in an elderly population [6]. In fact, it is projected that by 2025, 31.9% of Cameroonians (approximately 5.6 million people) will be living with hypertension [7].

Adherence to a medication regimen is generally defined as the extent to which patients take medications as prescribed by their health care providers [8]. Non-adherence is the main obstacle for controlling hypertension in the community, and a significant barrier to effective hypertension management [9–11]. Good adherence is therefore crucial to improve hypertension controls rates and prevent complications such as cerebrovascular accidents, coronary artery disease, aneurysms and heart failure [12, 13].

Several studies conducted globally on this topic have produced a wide range of results. The non-adherence rate in a global study conducted was 45% and a significant number of the hypertensive patients with co-morbidities were non-adherent to treatment [14]. The non-adherence rate from community-based studies in Bangladesh and Vietnam were as high as 85 and 49.8% respectively [15, 16]. About 66.7% of participants in a study conducted in both Nigeria and Ghana were non-adherent to treatment [17]. Similar studies conducted in Cameroon have reported low adherence rates of 43.9% [18] and 12.9% [19]. Several factors have been associated with non-adherence including but not limited to forgetfulness, lack of motivation due to the incurable nature of the disease, absence of symptoms, use of herbal preparation, physical disability, presence of complications, low level of education, poor knowledge of the disease and ignorace on the need for longterm treatment [17–20].

Hypertension accounts for a significant disease burden in Cameroon and control rates for blood pressure (BP) are poor. Adherence to medication is a key patient-factor in enhancing BP control, and community-based studies that have explored medication adherence in hypertensive populations in Cameroon are scant. We conducted this study to bridge the knowledge gap on the prevalence and factors associated with non-adherence to antihypertensive medication in Cameroon.

## Methods

### Study design and setting

This was a cross-sectional analytic study conducted from 12 November 2013 to 11 March 2014, a period of four months in the Buea Health District (BHD).

Buea is located at the eastern slope of the base of Mount Cameroon and is the capital of the South West Region. The BHD shares boundaries with Mount Cameroon to the west and north, Mutengene to the south, and Ekona to the east. Its population is estimated at 86,272 [21], and is made up of seven health areas including Buea road, Muea, Molyko, Bova, Buea Town, Bokwaongo and Tole.

### Sample size, sampling and eligibility

Using the following formula: $\boldsymbol{n}=\frac{\boldsymbol{p}\left(\mathbf{1}-\boldsymbol{p}\right){\boldsymbol{z}}^{\mathbf{2}}}{{\boldsymbol{d}}^{\mathbf{2}}}$

We estimated a minimum acceptable sample size (n) of 174 participants for this study. The standard normal variate for significance (z) was 1.96 and margin of error (d) set at 5%, and the prevalence (p) of adherence rate was considered to be 13% according to a community-based study conducted by Ekram et al. [22].

Participants were recruited using a two-staged cluster randomised sampling technique. The preliminary stage involved random selection through balloting to select four out of the seven aforementioned health areas. The Tole, Muea, Bokwaongo and Bova health areas were selected. With the permission of the local leaders, the objective of our study was channeled to the community through the local radio stations, focal person for communication, churches and meeting houses. This was followed by an exhaustive invitation of consenting eligible participants in every household. For every household, we inquired about the hypertension status of all adults who were 21 years and older. Participants who attested to have been diagnosed with hypertension and placed on treatment were required to state the name(s) and physically present the drug(s) they were taking for hypertension. This information was then confirmed with the participants’ hospital records. Only one participant was required per household. In case there was more than one person in a household who were eligible for this study, balloting was done to select the final participant to be included.

### Inclusion criteria

Consenting participants at least twenty-one years old living with hypertension who were on hypertensive medication(s) for at least one month were recruited for this study.

### Exclusion criteria

We excluded pregnant women, individuals who declared being hypertensive but had no proof that they were on or had been prescribed drugs, those who had smoked, or consumed alcohol or other cardiostimulants 30 min prior to data collection, and individuals who could not express themselves in either English or French.

### Data items and study procedure

Using a predesigned questionnaire, data was collected in two steps. First, through a one-on-one interview, data on socio-demographic characteristics (age, gender, marital status, religion, educational status, occupational status), clinical parameters (BP, duration of treatment, number of tablets and number of doses per day), level of adherence using the Morisky 8-item validated questionnaire [23], and factors potentially associated with non-adherence (like forgetfulness, absence of symptoms, use of herbal medicine, smell and taste of drugs, lack of funds to purchase drugs, busy schedule, and adverse drug effects) were collected. Second, the BP was measured respecting standardized protocol. For every participant, two BP readings were recorded at least 10 min apart using an automatic machine (Medical Rossmax-Automatic Upper Arm Blood Pressure Monitor, Model AK150f, manufactured by Rossmax International Ltd., Botzstrasse 6,D-07743 Jena, Germany) approximated to the nearest 1 mmHg. The average of both readings was then computed and used for analysis.

### Definition of operational variables

A controlled BP was defined as a systolic BP and diastolic BP below 140 mmHg and 90 mmHg, respectively.

Adherence to antihypertensive pharmacotherapy was defined as a Morisky score of ≤2 while non-adherence was defined as a Morisky score above 2.

Occupational status was categorised as low (require no expert training or no technical expertise like farming), medium (a technical expertise is required but no expert training e.g. carpentry, commercial bike riding and commercial taxi, etc.) and high (for professionals and require advanced training e.g. health personnel, teachers, accountants etc.).

### Ethical considerations

Ethical approval was granted by the Faculty of Health Sciences, Institutional Review Board (IRB) of the University of Buea (reference number: 2013/0106/UB/FHS/IRB) prior to conducting this study. In addition, administrative authorization was obtained from the South West Regional Delegation of Public Health before data collection.

### Data analysis

Data was entered in a Microsoft Excel 2007 spreadsheet and imported to the Statistical Package for Social Sciences (SPSS) version 20 software for analysis. Categorical variables were presented as frequencies and percentages. On the other hand, quantitative variables were reported as mean or median with their corresponding standard deviation (SD) or interquartile ranges (IQR), respectively. The chi squared or Fisher’s exact test and the student t-test were used for group comparisons for categorical and continuous variables, respectively. To determine predictors of non-adherence to antihypertensive medication, while adjusting for age, sex and other confounders, a multivariate logistic regression model using forward selection was built using variables with *p*-values below 0.25 on univariate analysis. The level of statistical significance was set at a p-value of 0.05.

## Results

### General characteristics

Table 1 portrays the socio-demographic and clinical characteristics of our study population. Out of 183 participants, 118 (64.5%) were females and the mean age was 55.9 ± 12.3 years. The ages of the participants ranged from 21 to 90 years. One hundred and twenty-six (68.9%) of the participants were married. One hundred and sixty-three (89.1%) were Christians. Majority (*n* = 139, 79.9%) of the participants had only up to primary school level of education. Ninety-five (51.9%) were employed.

**Table 1:** Characteristics of the study population

Variables	Category	Frequency (*n* = 183)	Proportion (%)
Age (years)	20–39	15	8.2
	40–59	89	48.6
	≥ 60	79	43.2
Gender	Male	65	35.5
	Female	118	64.5
Marital status	Single	17	9.3
	Married	126	68.9
	Divorced	3	1.6
	Wido*w*/widower	37	20.2
Religion	Christian	163	89.1
	Muslim	15	8.2
	None	5	2.7
Educational status	None	72	39.3
	Primary	67	36.6
	Secondary	31	16.9
	Tertiary	13	7.1
Employment status	Employed	95	51.9
	Unemployed	88	48.1
SBP (in mmHg) Mean (SD)			162.72 (26.1)
DBP (in mmHg) Mean (SD)			98.3 (17.7)
Daily dose frequency Median (IQR)			2(1–3)
Number of antihypertensive drugs Median (IQR)			1 (1–2)
Duration of treatment in years Median (IQR)			3 (2–4)

*SBP* Systolic blood pressure, *DBP* Diastolic blood pressure, *SD* Standard deviation, *IQR* Interquartile range

### Prevalence of non-adherence to antihypertensive pharmacotherapy

Out of 183 participants, 122 were non-adherent to their blood pressure lowering medication according to the Morisky medication adherence tool, giving a non-adherence prevalence of 66.7% (95%CI: 59.8–73.6%).

### Factors associated with non-adherence to antihypertensive pharmacotherapy

The following candidate variables were used in univariate analysis to test their association with non-adherence: multiple daily doses, multiple antihypertensive drugs, use of herbs, cost of treatment, absence of symptoms, forgetfulness, adverse drug effects, busy schedule. We found that forgetfulness (adjusted odds ratio (aOR) = 7.9; 95% confidence interval (CI): 3–20.8, *p* <  0.001), lack of finances (aOR = 2.8; CI: 1.1–6.9, *p* = 0.024), multiple daily doses (aOR =2.5; 1.2–5.6, p = 0.02) and drug side effects (aOR = 7.0 CI: 1.7–33.6, *p* = 0.007) were independent predictors of non-adherence after controlling for potential confounders in multivariate analysis. Our model, shown in Table 2, explained about 32.7% of the variability in the outcome variable (non-adherence)**.** When adjusted for age, gender, number of antihypertensive medications and duration on medications, participants who were adherent to medication were more likely to have good BP control (OR = 13.82; 95% CI = 5.46–34.95; *p* <  0.001), Table 3.

**Table 2:** Predictors of medication non-adherence among hypertensive patients in univariate and multivariate logistic regression

Variables	Non-adherent		OR	95% CI	*p*-value	AOR	95% CI	*p*-value
	Yes (*N* = 122)	No (*N* = 61)						
Age (in years) Mean (SD)	57.0 (12.3)	53.8 (12.2)	1.02	1.0–1.04	0.108	1.02	0.98–1.06	0.264
Gender								
Male	48 (39.3)	17 (26.2)	1.7	0.9–3.3	0.128	1.1	0.4–2.6	0.814
Female	74 (60.7)	44 (72.1)	Ref					
Marital status								
Single	11 (9.0)	6 (9.8)	Ref			–		
Married	86 (70.5)	40 (65.6)	0.9	0.3–3.0	0.857			
Divorced	2 (1.6)	1 (1.6)	0.8	0.4–1.6	0.489			
Widow/Widower	23 (18.9)	14 (23.0)	0.9	0.1–9.9	0.877			
Level of education								
None	47 (38.5)	25 (41.0)	Ref			–		
Primary	44 (36.1)	23 (37.7)	1.0	0.5–2.0	0.961			
Secondary	21 (17.2)	10 (16.4)	1.1	0.5–2.7	0.809			
Tertiary	10 (8.2)	3 (4.9)	1.8	0.4–7.0	0.415			
Employment status								
Unemployed	63 (51.6)	25 (41.0)	1.5	0.8–2.9	0.175	1.4	0.3–1.6	0.477
Employed	59 (48.4)	36 (59.0)	Ref					
Occupational status								
Low	38 (64.4)	23 (63.9)	0.8	0.3–2.5	0.735	–		
Medium	9 (15.3)	7 (19.4)	0.6	0.2–2.6	0.534			
High	12 (20.3)	6 (16.7)	Ref					
Daily dose frequency								
Mean (SD)	1.7 (0.6)	1.4 (0.6)	2.6	1.4–4.6	0.001*	2.5	1.2–5.6	0.021*
Number of antihypertensive								
Mean (SD)	1.7 (0.8)	1.3 (0.6)	2.5	1.4–4.3	0.001*	1.3	0.6–2.9	0.477
Duration of treatment (in years)								
Mean (SD)	3.1 (1.3)	2.8 (1.4)	0.2	0.9–1.5	0.215	0.9	0.7–1.3	0.590
Forgetfulness								
Yes	86 (72.3)	13 (21.7)	10.0	5.1–20.2	< 0.001*	7.9	3.0–20.8	< 0.001*
No	33 (27.7)	47 (78.3)	Ref			Ref		
Lack of funds								
Yes	67 (56.3)	18 (29.5)	3.3	1.7–5.0	0.001*	2.8	1.1–6.9	0.024*
No	52 (43.7)	43 (70.5)	Ref			Ref		
Busy schedule								
Yes	44 (37.0)	8 (13.3)	3.3	1.7–10.1	0.002*	2.0	0.6–6.5	0.247
No	75 (63.0)	52 (86.7)	Ref			Ref		
Experiencing side effects								
Yes	40 (33.6)	3 (4.8)	10.2	2.5–25.1	< 0.001*	7.6	1.7–33.0	0.007*
No	79 (66.4)	58 (95.1)	Ref			Ref		
Belief in the efficacy of the drug								
Yes	102 (85.7)	57 (95.0)	0.3	0.1–1.1	0.075	4.9	0.9–26.9	0.066
No	17 (14.3)	3 (5.0)	Ref			Ref		
Herbs								
Yes	25 (21.0)	13 (22.0)	1.1	0.5–2.3	0.875	–		
No	94 (79.0)	46 (78.0)	Ref					
Taste and smell of the drug								
Yes	1 (1.7)	3 (2.5)	0.7	0.1–6.5	0.717	–		
No	59 (98.3)	116 (97.5)						

*SD* Standard deviation, *OR* Odds ratio, *AOR* Adjusted Odd’s ratio, *CI* Confidence interval

*significant *p*-value; *N* = 183; df = 1, *p* <  0.001, R^2^ = 0.453

**Table 3:** Association between non-adherence to medication and blood pressure control^a^

Covariate	Blood pressure control status		OR (95% CI)	*P*-value
	Controlled (*N* = 40)	Uncontrolled (*N* = 143)		
Age in years, Mean (SD)	53.8 (14.0)	56.5 (11.8)	0.99 (0.96–1.04)	0.975
Gender (Male), N (%)	10 (25.0)	55 (38.5)	1.57 (0.63–3.90)	
Duration on treatment in years, Mean (SD)	2.75 (1.4)	3.06 (1.3)	0.86 (0.63–1.18)	0.353
Number of antihypertensive, mean (SD)	1.55 (0.7)	1.56 (0.6)	1.93 (0.98–3.77)	0.056
Adherence (Yes)	30 (75.0)	31 (78.3)	13.82 (5.46–34.95)	< 0.001**

*OR* Odd’s ratio, *SD* Standard deviation, *CI* Confidence interval

^a^Model adjusted for age, gender, number of antihypertensive drugs and duration of treatment; **Significant *p*-value; R^2^ = 0.328

## Discussion

In this community-based study assessing adherence to antihypertensive medication among adults in the BHD, we found that two-thirds of our study participants were non-adherent to antihypertensive pharmacotherapy and this was mainly driven by forgetfulness to take medications, lack of funds to buy medications, antihypertensive regimens requiring multiple daily dosing and drug side effects. In addition, non-adherence was associated with poor blood pressure control.

Two-thirds (66.7%) of our study population were non-adherent to treatment. This prevalence was similar to studies carried out in Ghana and Nigeria and Nigeria [17, 20], but higher than the values reported from studies in Czech Republic (31.5%), United Kingdom (41.6%) [24] and Canada (23%) [25]. These difference in the prevalence of non-adherence rates is probably due to better access to health care, availability of basic drugs for cardiovascular disorders in contrast to our setting where less than 60% of cardiovascular drugs are available [26], better standards of living with majority being insured and low illiteracy rates in these high income countries. In addition, we noted a higher non-adherence rate than the 13% prevalence rate obtained from a community-based study in Rajshashi in Bangladesh [22]. This discrepancy could be attributed to a difference in the methods used to assess non-adherence in both studies. In our study, the Morisky questionnaire was used to evaluate non-adherence while in the study done in Bangladesh, a participant was considered non-adherent if he/she missed his/her medication(s) on any day of the month. Furthermore, the high non-adherence rate in our study could be attributed to the fact that most of our study participants were just between 2 and 4 years of antihypertensive pharmacotherapy. Indeed, the adherence rate has been shown to increase with the duration on antihypertensive pharmacotherapy [27, 28].

After multivariable regression analysis, forgetfulness was significantly associated with non-adherence to antihypertensive pharmacotherapy. This is similar to the finding in Ghana where patients reported forgetfulness as the main reason for non-adherence [29]. The use of alarm clocks and/or education of other household members about when the drug(s) should be taken could improve adherence. Lack of finances was equally a predictor of medication non-adherence amongst our study population. Lack of finances appears to be a major predictor of non-adherence in sub-Saharan Africa [30–32] and other developing countries [33]. Unlike highly active antiretroviral drugs used in the management of the human immunodeficiency virus that are free, it is not the case with antihypertensive medications. These (hypertensive) patients need to buy their drugs out of their pockets. The absence of universal healthcare schemes and high unemployment rates in Africa [34] are chiefly responsible for the inability of hypertensive patients to afford their medications in a timely manner, and consequently achieve a good hypertensive control. Instituting a universal health insurance scheme is likely to improve access to, and utilization of healthcare facilities as well as drug availability.

People who experienced side effects of the drugs were more likely to be non-adherent than their counterparts. This corroborates with the findings of Okoro and Ngong in Nigeria whereby, feeling worse (side effects of antihypertensive drugs) and feeling better were independent predictors of non-adherence [35]. This finding is supported by those of other sub-Saharan countries [36] and elsewhere [37]. Health care providers need to devote time in educating patients about this chronic disorder and its management. Affected individuals should be enlightened on the need to report to the hospital in case of any inconveniences posed by the drug rather than personally stopping the drugs without medical advice. Patients should also be given regular appointments for proper follow up and those who miss their appointments should be contacted or strategies put in place to ensure retention in care and limit loss to follow-up.

Evidence on the association between multiple factors including age and gender, and non-adherence to antihypertensive medication is controversial and warrants careful interpretation [11, 28]. Caro [38] and Gupta [24] found that females were more likely to be non-adherent to antihypertensive pharmacotherapy, contrary to the report of other authors in which males were more adherent to treatment than females [39–41]. On the other hand, some authors have reported no association between gender and adherence rate [42–44]. In a recent global meta-analysis on non-adherence to antihypertensive pharmacotherapy assessed using the MMAS-8 tool, Abegaz et al. reported that males were 1.3 times more likely to be non-adherent after a gender subgroup analysis, however their findings were not statistically significant [45]. Generally, younger age has been associated with greater odds of non-adherence in a number of studies [24, 42, 44], whereas in line with our findings, others have revealed no significant association [46]. These discrepancies with age as a predictor of non-adherence to antihypertensive pharmacotherapy could be attributed, in part, to the dichotomization of age, which results in loss of statistical power and precision [39, 47]. Furthermore, failure to explore this association using a multivariable logistic regression analysis to account for potential confounders [39, 42], differences in the technic or tool used to assess medication adherence [48] or the difference in sample sizes across studies are other alternate explanations.

Non-adherence was associated with poor blood pressure control. This finding accords with several results obtained in other countries in sub-Saharan Africa [17] and elsewhere [46, 49, 50]. The importance of adhering to medication cannot be overemphasized in the fight to prevent hypertension-related morbidity and mortality, as adequate treatment and control of BP has been highlighted among the strategies to reduce burden of CVD by 25% by the year 2025 [51]. However, this finding disagrees with that from other sub-Saharan countries [27] which could be attributed to a difference in study setting (community- versus hospital-based) and frequency of BP measurements (single versus multiple BP measures). For example, Mekonnen et al. 2017 [27] measured the BP just once. The average of multiple BP readings is critical to reduce type 1 error.

### Strengths and limitations

Our study had a number of limitations. First, our cross-sectional design precluded assessment of temporality; rather we could only obtain associations. Second, only a single questionnaire was used to assess adherence in this study. The use of a second scale to measure medication adherence, such as the Medication Adherence Rating Scale would potentially have improved on the reliability of our findings. Third, pathologies such as chronic kidney disease, which can influence blood pressure control and indirectly adherence rates, were not excluded. Fourth, all seven health areas of this district could not be sampled due to financial restrictions, which limited a more complete picture on antihypertensive medication non-adherence in this health district. However, the authors believe that with a random selection of four out of the seven health areas gave each health area a fairly equal probability to be included. Fifth, participants on antihypertensive pharmacotherapy for just a year were recruited in this study. This could be a source of bias. Indeed, shorter treatment durations have been associated with higher non-adherence rates. In addition, predictors of medication non-adherence such as a poor patient-provider relationship was not captured in this study [11]. The Morisky medication adherence scale (MMAS) is largely based on self-report, making it liable to recall bias. Additionally, this can potentially lead to misclassification with individuals providing inaccurate responses. Despite these shortcomings, the MMAS has been widely validated in patients with hypertension [23] and other chronic diseases [52] with fairly good performance (sensitivity of 93% and specificity of 53%) in the clinic setting. It is thus the most widely accepted self-report tool for assessing medication adherence, especially to detect medication non-adherence with good blood pressure control data [23].

## Conclusions

Two-thirds of our study population were non-adherent to antihypertensive pharmacotherapy. Forgetfulness, multiple daily doses, lack of finances, and side effects of drugs were associated with non-adherence. Non-adherence to medication was associated with uncontrolled blood pressure. The use of reminders like alarms, prescription of generic drugs, less complex regimens and intensive patient/carer education are of paramount importance in tackling these factors. Lessons from interventions promoting retention in care and medication adherence among persons living with HIV could be applied for hypertensive populations. In addition, institution of a universal insurance scheme is likely to improve patient accessibility to healthcare facilities and the availability of antihypertensive drugs.

## Abbreviations

BHD: Buea Health District
BP: Blood pressure
CVD: Cardiovascular disease
HIV: Human immunodeficiency virus
LMIC: Low-income and middle-income countries
MMAS: Morisky medication adherence scale
OR: odds ratio
